# 直接测序法和ARMS法检测非小细胞肺癌表皮生长因子受体基因突变的比较

**DOI:** 10.3779/j.issn.1009-3419.2014.08.05

**Published:** 2014-08-20

**Authors:** 辰晨 李, 建中 吴, 卓 王, 继锋 冯

**Affiliations:** 1 210009 南京，南京医科大学附属江苏省肿瘤医院临床肿瘤实验中心 Clinical Oncology Center, Jiangsu Cancer Hospital, Affiliated to Nanjing Medical University, Nanjing 210009, China; 2 210009 南京，南京医科大学附属江苏省肿瘤医院内科 Internal medicine, Jiangsu Cancer Hospital, Affiliated to Nanjing Medical University, Nanjing 210009, China

**Keywords:** *EGFR*基因突变, 直接测序法, ARMS法, 肺肿瘤, *EGFR* mutation, Direct sequencing, ARMS, Lung neoplasms

## Abstract

**背景与目的:**

以表皮生长因子受体(epidermal growth factor receptor, EGFR)为靶点治疗非小细胞肺癌(non-small cell lung cancer, NSCLC)是现在治疗肺癌的前沿手段, 因此检测EGFR是否突变成为治疗肺癌的关键一步。本研究旨在探讨直接测序法和ARMS法检测NSCLC患者的*EGFR*基因突变情况及检出率。

**方法:**

收集自2012年4月-2013年6月本中心接受进行*EGFR*基因突变检测的NSCLC患者, 分别用直接测序法和ARMS法对这些患者的肿瘤组织标本进行检测, 检测其中*EGFR*基因第18-21外显子的突变情况, 并比较两者方法的优劣。

**结果:**

在该451例两种方法均检测的患者中, 两种方法均检测到突变且突变结果一致者127例, 结果不一致者5例, 均无突变者186例, 直接测序法检测到突变而ARMS法未检测到突变者50例, 反之83例。50例中有33例为ARMS法29种突变之外的突变。直接测序法检测的突变率为40.4%, ARMS法检测的突变率为47.7%, ARMS法的突变检出率明显高于直接测序法(*P* < 0.001)。在204例石蜡组织中, ARMS法的突变检出率59.80%明显高于直接测序法41.67%(*P* < 0.001);而在240例新鲜组织中, 两种方法无统计学差异(*P*=0.083)。

**结论:**

直接测序法和ARMS法检测*EGFR*基因突变基本一致, ARMS法更为灵敏, 且操作方便快捷, 但是价格昂贵。对于肿瘤组织含量较少的样本中, ARMS法更为敏感, 明显优于直接测序法。直接测序法可以检测到ARMS试剂盒内不包含的少见突变。结合两种方法检测结果更为可靠全面。

## Abstract (en)

**Background and objective:**

Epidermal growth factor receptor (EGFR) inhibitors are mainly used for the targeted therapy of non-small cell lung cancer (NSCLC).Therefore, *EGFR* mutations should be detected to treat lung cancer.The aim of this study is to determine the detection rate of NSCLC in patients with *EGFR* gene mutations by conducting direct sequencing and ARMS assay.

**Methods:**

A total of 451 patients who were diagnosed with NSCLC between April 2012 and June 2013 participated in this study.Gene mutation was detected in the exon of *EGFR* 18 to 21 by direct sequencing and ARMS assay.

**Results:**

All of the 451 cases of NSCLC were subjected to direct sequencing and ARMS assay.Using both techniques, we detected the same *EGFR* mutation in 127 cases and different *EGFR* mutations in 5 cases, but no mutations were detected in 186 cases.In direct sequencing alone, *EGFR* mutation was detected in 50 cases.In ARMS assay alone, *EGFR* mutation was detected in 83 cases.The mutation rates of direct sequencing and ARMS assay were 40.4% and 47.7%, respectively.Therefore, the mutation detection rate of ARMS assay was significantly higher than that of direct sequencing (*P* < 0.001).In 204 paraffin tissue samples of NSCLC, the mutation detection rate of ARMS assay (59.80%) was significantly higher than that of direct sequencing (41.67%;*P* < 0.001).By comparison, the mutation detection rates of ARMS assay (39.58%) and direct sequencing (38.33%) showed no significant difference (*P*=0.083) when 240 fresh tissue samples of NSCLC were used.

**Conclusion:**

Direct sequencing and ARMS assay exhibited similar efficacy in detecting *EGFR* mutations.Despite its high operational costs, ARMS assay was more sensitive and more convenient than direct sequencing, particularly when a small number of tissues were used.By comparison, direct sequencing could detect mutations that were not detected by ARMS assay.Therefore, the combination of direct sequencing and ARMS assay could provide more reliable and comprehensive test results than the lone application of each technique.

肺癌是死亡率较高的一种恶性肿瘤, 世界范围内每年因肺癌而死亡的人数大约有100万^[1]^。目前在肺癌的治疗策略方面取得了一定的进步, 但患者5年生存率仍然只有15%^[2]^。随着近几年发现在非小细胞肺癌(non-small cell lung cancer, NSCLC)中含有表皮生长因子受体(epidermal growth factor receptor, *EGFR*)基因突变的比例增高, 肺癌的治疗策略也随之发生了巨大变化^[3, 4]^。酪氨酸激酶抑制剂(tyrosine kinase inhibitors, TKIs)能够特异性地抑制突变的EGFR蛋白, 如吉非替尼和厄洛替尼。

在2011年中国版有关NSCLC的美国国家综合癌症网络(National Comprehensive Cancer Network, NCCN)指南中, 根据最新的Ⅲ期随机研究, 如IPASS、First-SIGNAL、WJTOG3405、OPTIMAL治疗, TKIs已作为一线治疗方案, 并且*EGFR*活化突变的存在对一些合适的患者的选择是一个关键的生物学因素^[5-11]^。因此, 在中国很多医院, *EGFR*突变分析已成为一种常规的分子检测项目。并且直接测序法是最常用的的方法, 因为易获得, 与实时PCR分析法(如TaqMan探针)、ARMS法和HRM法相比, 直接测序法相对价格便宜。

众所周知, 肿瘤组织是*EGFR*突变分析的最佳DNA资源。然而, 大多数NSCLC患者处于晚期不能手术, 足够的肿瘤组织不易获得。例如, 在IPASS研究中, 只有36%(437/1, 217)患者有组织活检标本适用于检测。在INTEREST研究中, 这个比例仅为20%(297/1, 466)^[5, 12]^。相反, 体液的获取通常很容易, 创伤小, 且可重复, 例如胸水和血浆^[13-18]^。然而, 使用体液的突变的测试程序还需要进行优化、标准化和验证的。

本研究通过直接测序法和扩增阻滞突变系统(amplification refractory mutation system, ARMS)方法(ADx-EGFR 29, 厦门艾德公司)来比较不同来源的肿瘤组织标本(包括新鲜组织、石蜡组织及胸水)的*EGFR*基因中18-21外显子的突变情况。

## 1 材料和方法

### 1.1 材料

收集自2012年4月-2013年6月本中心接受进行*EGFR*基因突变检测的NSCLC患者。这些患者肿瘤组织标本分为新鲜组织、石蜡组织和胸水组织, 主要来源于手术切除、穿刺活检以及胸水脱落细胞。

### 1.2 方法

新鲜及胸水标本先加入裂解液, 从400 μL裂解液中用试剂盒(Qia-gen, Hilden, Germany)提取DNA, 再用50 μL的蒸馏水洗脱, 最后将提取好的DNA放入-20 ℃冰箱保存备用。提供的石蜡组织, 切10张-15张白片, 同时染1张HE切片, 并在HE切片上标记肿瘤组织区, 将白片用无水乙醇浸泡后, 对照HE切片刮取收集肿瘤组织, 加入裂解液, 从400 μL裂解液中用试剂盒(Qia-gen, Hilden, Germany)提取DNA, 再用50 μL的蒸馏水洗脱, 最后将提取好的DNA放入-20 ℃冰箱保存备用。通过聚合酶链反应(Polymerase Chain Reaction, PCR)将*EGFR*基因中18-21外显子用特异性引物进行扩增。最终的扩展分别用直接测序法和ARMS法来比较两者的测试结果。对于两个方法测试结果不一致的, 先进行复测, 复测后仍不一致者采用ADX-EGFR试剂盒重新测试。

直接测序法是将提取的产物与特异性引物用ABI PRISM 3730 DNA分析器进行分析测序(应用生物系统, CA, USA)。ARMS法是将患者的DNA用ADx-*EGFR*突变检测试剂盒检测, 该试剂盒利用扩增阻滞突变系统(ARMS)的原理, 涵盖了*EGFR*基因中18-21外显子的29个突变点, 包括3种18外显子突变, 19种19外显子突变, 5种20外显子突变和2种21外显子突变。

### 1.3 统计学分析

所有统计学检验采用SPSS 18.0统计软件进行数据分析, *P* < 0.05为差异有统计学意义。

## 2 结果

### 2.1 患者和样本特征

自2012年4月-2013年6月本中心接受进行*EGFR*基因突变检测的患者共451例, 分别用直接测序法和ARMS法检测。这些患者包括204例男性, 247例女性, 平均年龄54.8岁±9.5岁, 年龄跨度32岁-88岁。新鲜组织包括手术切除、纤维支气管镜活检、肺及淋巴结穿刺的新鲜标本; 石蜡组织包括除了胸水之外的病理标本经过处理后的标准FFPE标本; 胸水为胸水脱落细胞。有肿瘤组织学分型的患者有406例, 其中鳞癌57例, 腺癌329例, 其他20例。

### 2.2 直接测序法检测结果

451例患者使用直接测序法检测, 其中有182例检测到*EGFR*基因突变, 突变率为40.4%(182/451);单突变178例, 包括18外显子7例, 占所有突变的3.9%(7/178);19外显子99例, 占所有突变的55.6%(99/178);20外显子4例, 占所有突变的2.3%(4/178);21外显子68例, 占所有突变的38.2%(68/178), 主要是L858R点突变。另外检测到双突变2例, 1例为G724S+L858R;1例为S768I+L858R。三突变2例, 分别为L730F+S768I+21外显子点突变; 19外显子插入突变+S768I+L858R。

### 2.3 ARMS法检测结果

451例患者使用ARMS法检测, 其中有215例*EGFR*基因突变, 突变率为47.7%(215/451), 单突变212例, 其中18外显子4例, 占所有突变的1.9%(4/212);19外显子突变117例, 占所有突变的55.2%(117/212), 主要是缺失突变; 20外显子1例, 占所有突变的0.5%(1/212);21外显子90例, 占所有突变的42.4%(90/212), 主要是L858R点突变。另外检测到双突变3例, 为S768I+L858R双突变。

### 2.4 直接测序法和ARMS法检测结果比较

在该451例两种方法均检测的患者中, 两种方法均检测到突变且突变结果一致者127例, 结果不一致者5例, 均无突变者186例, 直接测序法检测到突变而ARMS法未检测到突变者50例, 反之83例。50例中有33例为ARMS法29种突变之外的突变。根据表 3所示, 直接测序法和ARMS法的诊断符合率为70.51%(318/451)。

**3:** 直接测序法和ARMS法检测结果比较 The comparison between direct suquencing and ARMS

	Direct suquencing (182)				
			+	-	Total
ARMS (215)	+		132	83	215
	-		50	186	236
Total			182	269	451

**1:**
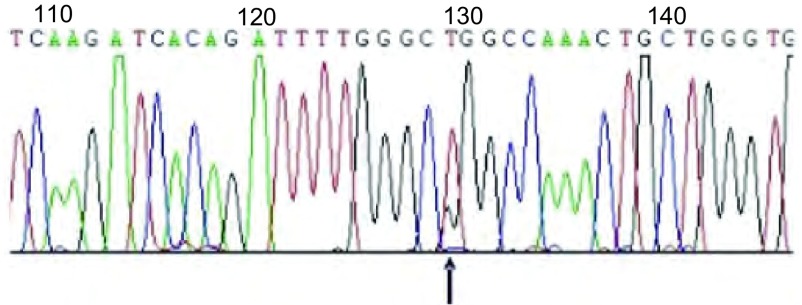
直接测序法检测21外显子L858R点突变 Exon 21 L858R point mutation by direct sequencing

**2:**
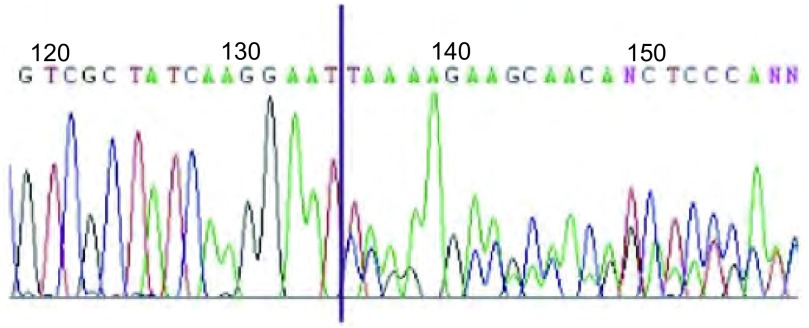
直接测序法检测19外显子缺失突变 Exon 19 eletion mutation by direct sequencing

### 2.5 不同组织来源检测结果

在该451例患者中, 新鲜组织240例, 石蜡组织204例, 胸水7例。240例新鲜组织中, 直接测序法检测到突变者95例, 突变率为39.58%(95/240), ARMS法检测到突变者为92例, 突变率为38.33%(92/240);两种方法突变率无统计学差异(*P*=0.083)。在204例石蜡组织标本中, 直接测序法检测到突变者85例, 突变率为41.67%(85/204), ARMS法检测到突变者为122例, 突变率为59.80%(122/204);两种方法突变率有统计学差异(*P* < 0.001)。在7例胸水组织中, 直接测序法检测到突变者2例, 突变率为28.57%(2/7), ARMS法检测到突变者为1例, 突变率为14.29%(1/7);但由于胸水组织标本量过少, 不具代表性, 在此不进行统计学分析。

## 3 讨论

在NSCLC的治疗中, 分子靶向药物TKIs的应用开启了肺癌治疗的新道路, 现可作为一线治疗。TKIs疗效好, 针对性强, 副作用小, 但价格昂贵, 对于该药敏感性的检测就是决定是否可以用药的关键。目前公认最有效的预测TKIs疗效的生物标志为EGFR激酶区的突变, EGFR突变与TKIs对于NSCLC患者的治疗效果是紧密联系在一起的, 因此临床上需要一种快速、灵敏、准确的检测*EGFR*突变的方法。

直接测序法应用PCR直接扩增*EGFR*基因第18-21外显子的基因片段, 目前普遍认为是*EGFR*基因突变检测的金标准。但其过程比较繁琐, 耗时长; 对标本所含肿瘤组织的要求比较高, 敏感性不低于30%突变拷贝数^[19]^。测序峰中是否出现了突变波决定了PCR结果的判断, 因此, 突变波波峰的高低影响判断的结果。在本研究中, 对于直接测序法269例未检出突变的患者中, ARMS法多检测出了50例, 主要为21外显子突变, 该研究表明在直接测序法中, 21外显子较其他外显子更易漏检。

**1:** 直接测序法检测出的33例ARMS试剂盒不包括的突变 33 mutations by direct sequencing that ARMS kit not include

Number	Direct suquencing	ARMS	Organization type
1	G724S	No mutations	Fresh tissue
2	G2656C	No mutations	Fresh tissue
3	G2656C	No mutations	Fresh tissue
4	2127-2129 Del	No mutations	Fresh tissue
5	2203-2204 Ins	No mutations	Fresh tissue
6	2205-2206 Ins	No mutations	Fresh tissue
7	2205-2206 Ins	No mutations	Paraffin tissue
8	2205-2206 Ins	No mutations	Paraffin tissue
9	2205-2206 Ins	No mutations	Paraffin tissue
10	2205-2206 Ins	No mutations	Paraffin tissue
11	2206-2207 Ins	No mutations	Fresh tissue
12	K754E	No mutations	Fresh tissue
13	E758K	No mutations	Paraffin tissue
14	E758G	No mutations	Paraffin tissue
15	E746K	No mutations	Paraffin tissue
16	P753S	No mutations	Fresh tissue
17	L747L (no meaning)	No mutations	Fresh tissue
18	L747S	No mutations	Fresh tissue
19	2252-2253 Del	No mutations	Fresh tissue
20	2239-2252 Del	No mutations	Fresh tissue
21	2239-2249 Del	No mutations	Paraffin tissue
22	2237-2253 Del	No mutations	Paraffin tissue
23	2237-2253 Del	No mutations	Fresh tissue
24	2236-2245 Del	No mutations	Fresh tissue
25	2238-2256 Del	No mutations	Fresh tissue
26	exon 19 mutation	No mutations	Fresh tissue
27	2300-2306 Ins	No mutations	Fresh tissue
28	exon 20 mutation	No mutations	Fresh tissue
29	exon 20 mutation	No mutations	Fresh tissue
30	Q849R	No mutations	Paraffin tissue
31	C2156T	No mutations	Paraffin tissue
32	V835L	No mutations	Paraffin tissue
33	L861R	No mutations	Pleural effusion organization
ARMS:amplification refractory mutation.			

**2:** 两种方法检测的不一致的突变 Inconsistent mutations by the two methods

Number	Direct suquencing	ARMS	Organization type
1	L858R	S768I+L858R	Paraffin tissue
2	L858R	S768I+L858R	Paraffin tissue
3	19 Del	L858R	Paraffin tissue
4	19 Del	L858R	Fresh tissue
5	19 Ins+S768I+L858R	S768I+L858R	Fresh tissue

ARMS法是利用Taq DNA聚合酶针对不同的已知突变, 设计适当的引物以检测出突变基因。扩增产物通过实时荧光定量PCR技术进行分析。与直接测序法相比, ARMS法敏感度高, 为1%^[20]^, 检测起来方便快捷。但ARMS法仅适用于单个已知基因的突变, 且由于仪器昂贵限制了临床应用。Kimura等^[21]^和Horiike等^[22]^先后报道了ARMS法的高度特异性和灵敏度, 但他们仅仅检测了两个最常见的突变位点—第19外显子DelE746-A750和第21外显子L858R, 所检测的其他突变类型也较局限, 且可能出现漏检。在本研究中, 通过直接测序法检测出来ARMS试剂盒29种突变之外的突变为33例, 表明ARMS法仅可检测出常见突变, 也可能出现漏检。但是对于29种突变之外的突变, 是否对TKIs有一定的疗效, 还有待进一步的研究。

从不同肿瘤组织的样本取材来看, 不同组织对于*EGFR*突变检测的结果也有一定的影响。许多有关直接测序法和ARMS法检测*EGFR*基因突变的研究都已表明ARMS法比直接测序法具有更高的特异性和灵敏度。赵婧雅等^[23]^的研究表明, 对于活检小标本而言, ARMS法较直接测序法拥有更高的突变检出率; 而对于手术标本无统计学差异。在本研究中, 新鲜组织中含有较多的肿瘤组织, 因此两者比较无明显统计学差异; 而对于肿瘤组织较少的样本, 需要较高敏感度, ARMS法则明显优于直接测序法。而对于胸水组织样本的比较以往也有研究, 但本例样本量少, 不予讨论。因此, 可以根据不同组织来源的样本, 选择合适的检测方法, 从特异性、敏感性、经济快捷各方面取得最优方案。

本研究中也有很多不足之处, 在该451例患者中, 胸水组织样本过少, 不具代表性, 不能进行统计学分析。另外, 本研究中的ARMS法敏感性较其他研究文献中偏低, 原因可能是有相当比例的标本是新鲜组织, 标本的一部分用直接测序法检测, 一部分用ARMS法检测, 检测前并不能确定其中是否含有肿瘤组织, 这也就导致两种方法检测的不一致。较理想的做法是经病理确认, 同一份DNA分成二份来分别检测。最后, 检测的目的是指导靶向药物的治疗, 但由于经济等综合原因, 只有少数患者在本中心选择靶向治疗, 其数据和结果也不具备一定的代表性, 在此也未予讨论。

直接测序法和ARMS法检测*EGFR*基因突变基本一致, ARMS法更为灵敏, 且操作方便快捷, 但是价格昂贵; 对于肿瘤组织含量较少的样本中, ARMS法更为敏感, 明显优于直接测序法; 直接测序法可以检测到ARMS试剂盒内不包含的少见突变, 结合两种方法检测结果更为可靠全面。
